# Dosimetric Study of Heat-Treated Calcium–Aluminum–Silicon Borate Dosimeter for Diagnostic Radiology Applications

**DOI:** 10.3390/s23021011

**Published:** 2023-01-16

**Authors:** Ibrahim Algain, Mehenna Arib, Said A. Farha Al-Said, Hossam Donya

**Affiliations:** 1 Physics Department, Faculty of Science, King Abdulaziz University, Jeddah 21589, Saudi Arabia; 2 Health Physics Section, King Faisal Specialist Hospital and Research Centre, Riyadh 11564, Saudi Arabia; 3 Secondary Standard Dosimetry Laboratory, King Faisal Specialist Hospital and Research Centre, Riyadh 11564, Saudi Arabia; 4 Department of Physics, Faculty of Science, Menoufia University, Shibin El-Koom 32511, Egypt

**Keywords:** thermoluminescence radiation dosimeter, glass-ceramic dosimeters, radiology dosimetry, low dose

## Abstract

The production of thermoluminescence (TL) dosimeters fabricated from B_2_O_3_-CaF_2_-Al_2_O_3_-SiO_2_ doped with Cu and Pr for use in diagnostic radiology is the main goal of this research. The TL samples were synthesized via the melt-quench technique processed by melting the mixture at 1200 °C for 1 h, and, after cooling, the sample thus created was divided into two samples and retreated by heating for 2 h (referred to as TLV30) and for 15 h (referred to as TLV17). SEM and EDS analyses were performed on the TL samples to confirm the preparation process and to investigate the effects of irradiation dosimetry on the TL samples. Furthermore, the TL samples were irradiated with γ-rays using a 450 Ci ^137^Cs irradiator and variable X-ray beams (5–70 mGy). Two important diagnostic radiology applications were considered: CT (6–24 mGy) and mammography (2.72–10.8 mGy). Important dosimetric properties, such as the glow curves, reproducibility, dose–response linearity, energy dependence, minimum dose detectability and fading, were investigated for the synthetized samples (TLV17 and TLV30), the results of which were compared with the Harshaw TLD-100. The TLV17 dosimeter showed higher sensitivity than TLV30 in all applied irradiation procedures. The dose–response linearity coefficients of determination R^2^ for TLV17 were higher than TLD-100 and TLV30 in some applications and were almost equal in others. The reproducibility results of TLV17, TLV30 and TLD-100 were less than 5%, which is acceptable. On the other hand, the results of the fading investigations showed that, in general, TLV17 showed less fading than TLV30. Both samples showed a significant decrease in this regard after the first day, and then the signal variation became essentially stable though with a slight decrease until the eighth day. Therefore, it is recommended to read the TL dosimeters after 24 h, as with TLD-100. The SEM images confirmed the existence of crystallization, whilst the EDS spectra confirmed the presence of the elements used for preparation. Furthermore, we noticed that TLV17 had grown dense crystals that were larger in size compared to those of TLV30, which explains the higher sensitivity in TLV17. Overall, despite the fading, TLV17 showed greater radiation sensitivity and dose–response linearity compared with TLD-100. The synthetized TL samples showed their suitability for use as dosimeters in diagnostic radiology radiation dosimetry.

## 1. Introduction

Thermoluminescence (TL) dosimetry is used in many applied fields, such as medical physics, radiation protection, industry, environmental and cosmic radiation, with many different materials. In general, a TL material used for dosimetry should demonstrate good dose linearity, high radiation sensitivity, good reproducibility and low fading [1,2,3].

Thermoluminescence dosimeter (TLD) material comes in a variety of shapes and sizes, including crystals, powder and pellets. Unfortunately, none of the currently available TLD meet all of the criteria for a perfect dosimeter. Lithium fluoride doped with magnesium and titanium (LiF: Mg, Ti) (TLD-100) [4] is the most widely used TL phosphor; however, it suffers from significant drawbacks [5,6]. It is partially soluble in water [7] and has a tendency to vary in sensitivity with use [8].

In the past few years, many materials with different components were studied using radiation dosimetry. Recent studies on TL glasses or ceramics containing one or more components CaF_2_, Al_2_O_3_, B_2_O_3_ and SiO_2_ were performed. A study on Li_2_O-B_2_O_3_ doped with Ag showed glow curves at temperatures of 220 and 320 °C and a higher response than undoped ones. Silver atoms (105 ppm) doped with lithium borate glasses irradiated by γ-rays showed linearity over the studied dose-range 1–70 Gy [9]. 

In 2019, a study on CaF_2_-Al_2_O_3_-B_2_O_3_ doped with CeF_3_ showed glow curves at temperatures of 125 and 360 °C and a higher glow curve when doped with 0.75% and 2% CeF_3_. The linearity was from the dose range of 10 to 1000 mGy in undoped samples and 0.01 to 1000 mGy for doped samples [10]. A study on a glass system of Sm_2_O_3_-doped BaO–ZnO–LiF–B_2_O_3_ irradiated by γ-rays showed good results in industrial applications, specifically in food irradiation. The dose range was linear between 0.25 and 3 kGy, showed high sensitivity with a minimum dose detection of 2 Gy and further showed reproducibility and reusability after annealing at a temperature of 250 °C [11]. 

Another investigation performed on a glass sample of ZrO_2_ doped with Mg for radiation therapy dosimetry saw the samples exposed to 1 Gy using a 6 MV linac X-ray beam. The results were found to fade by 47% after 24 h, and this was not appropriate for radiotherapy dose measurements because of the low sensitivity to multiple doses, low temperature for the TL peak, low reproducibility and significant fading [12]. The Al-doped LiB_3_O_5_ nanophosphor exhibited a linear dose-response and a smooth energy response over the 0.01–100 Gy γ-exposure range. After two months, it had faded by less than 10 percent. 

In medical applications, the TL nanophosphor was confirmed to be a promising material for gamma dosimetry [13]. A TL study performed on Y_2_O_3_:Sm^+3^ nanophosphor irradiated by ^60^Co γ-rays only showed a dose response and can be used for high dose gamma applications only [14]. Another work was proposed, a glass system composed of B_2_O_3_, Gd_2_O_3_, Bi_2_O_3_ and Li_2_O doped with cerium fluoride, CeF_3_, where this dosimeter demonstrated a linear radiation dose response between 10 and 100 mGy [15]. Other applications were investigated by our group using glassy materials [16,17] for various medical applications.

The main goal of the present research is to study the properties of a thermoluminescent dosimeter prepared from heated-treated composite B_2_O_3_-CaF_2_-Al_2_O_3_-SiO_2_ doped with copper (Cu) and presidium (Pr), to determine its ability to detect gamma and X-rays in the 2–70 mGy dose range used in diagnostic radiology and to further compare this system with Harshaw TLD-100 to determine if its properties are preferable in use. The effects of forming crystals in a glass system of amorphous state resulting from the thermal treatment of samples on the physical properties was investigated in our previous work [18]. However, no previous study had been performed or fully tested using diagnostic radiology applications for this particular composition. 

Well-established medical imaging applications—namely, CT and mammography—were used to examine the synthesized TL samples. Although CT is well-known for its advantages in precise diagnosis, these are not without potential risk. In comparison to other traditional radiation treatments, CT has a significantly higher patient dose [19]. When compared to other radiography systems, the mammography unit operates in a low tube voltage range, often between 25 and 35 kVp; however, a slight change in its operating parameters might result in the delivery of larger doses, which can be harmful because the breast is a radiosensitive organ. Therefore, it is crucial to ensure that the machine’s operating parameters are up to standard and are maintained [20]. 

The entrance skin dose and Average Granular Dose (AGD) are important quantities that should be measured in mammography QC making sure that unnecessary radiation doses to patients is avoided. AGD should be less than 3 mGy per image in 2D or Tomography (3D) [20].

## 2. Materials and Methods

### 2.1. TL Dosimeter Synthesization

A melt-quench technique was used for the preparation of the TL dosimeters. The preparation proceeded via two stages: the first was to prepare four host elements with 10%CaF_2_:10% SiO_2_, 10%Al_2_O_3−_70%B_2_O_3_ (purity of 99–99.8%). The doping elements had an equal weight (1500 ppm) of two oxides: one is rare-earth Pr_2_O_3_, and the other is transition metal CuO. At the beginning, a platinum crucible contain the abovementioned mixture was placed in an oven at a temperature of 350 °C for two hours, and then it was moved to a controllable furnace under natural atmospheric conditions and at a temperature of 1200 °C for one hour. 

**Figure 1:**
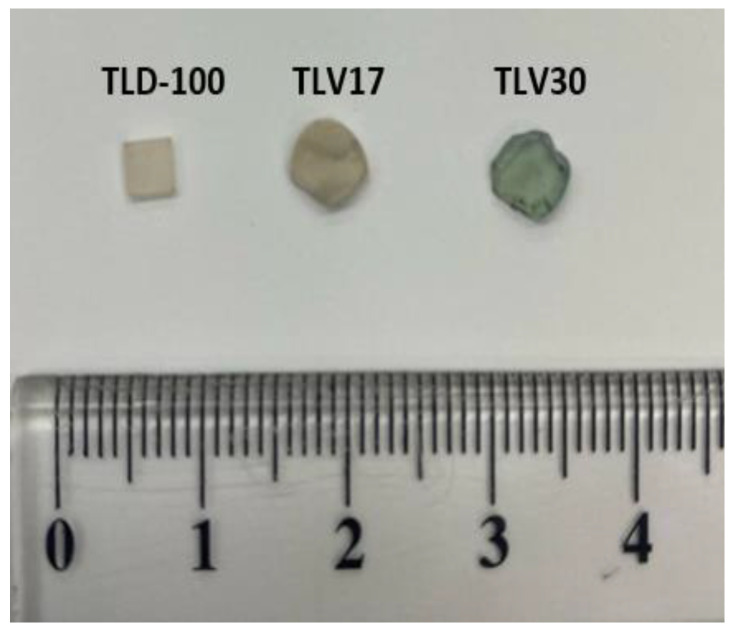
The synthesized TL samples TLV17, TLV30 and Thermofisher TLD-100.

Then, the homogenous melt was poured into a heated stainless steel mold to make cylindrical shaped glass samples with a diameter 2–3 mm and a height of 2 mm, which were then quickly moved to an annealing furnace. The samples were annealed for two hours at 350 °C (below the glass transition temperature T_g_). The furnace was switched off to cool down and reach room temperature (see Table 1). In the second stage, the synthesized composition was divided into two samples. The first sample was thermally treated again for 2 h at temperature 550 °C (between the T_g_ and crystalline (T_c_) temperatures), referred to as TLV30, whilst the second TL sample was treated for 15 h at the same temperature, referred to as TLV17 (see table and Figure 1).

### 2.2. Scanning Electronic Microscopy (SEM) and Energy Dispersive X-ray Analysis (EDS)

To confirm the existence of the elements used (hosts and dopants) for the TL dosimeters prepared in this study, SEM (Model JSM-6380LA equipped with an Oxford Instruments EDS system X-Max with a resolution of 5.9 at 127 eV, running the Aztec software) was performed on the samples. Analyses were conducted for sixty seconds at an accelerating voltage of 15 kV with a beam current of approximately 70 pA [21].

### 2.3. Irradiation Facilities

#### 2.3.1. Secondary Standard Dosimetry Laboratory (SSDL)

**Figure 2:**
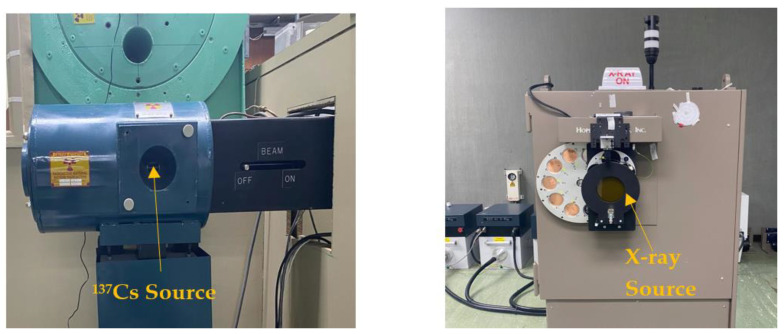
KFSH&RC-SSDL 450 Ci 137Cs and X-ray sources.

The prepared samples were irradiated at the SSDL of the Biomedical Physics Department (BPD), King Faisal Specialist Hospital and Research Centre (KFSH&RC) [22]. Irradiations were performed using a 450 Ci ^137^Cs source of γ-rays (see Figure 2) delivering radiation doses of 5, 10, 20, 30, 40, 50, 60 and 70 mGy. In addition, they were irradiated by SSDL diagnostic radiology X-ray beams at doses of 5, 10, 20, 30, 40, 50, 60 and 70 mGy at different tube voltages of 40, 80, 100, 120 and 150 kVp with effective energies of 26.1, 36.3, 41.8, 47.3 and 61 keV, respectively [23].

#### 2.3.2. Mammography

**Figure 3:**
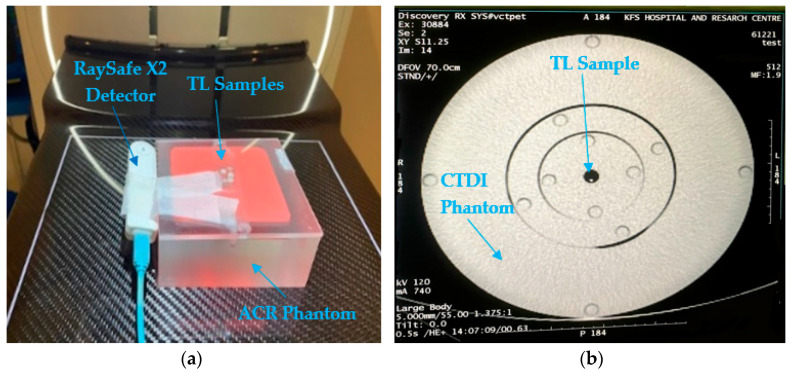
(**a**) TLD irradiation from mammography and (**b**) CT image of TLV17 and TLV30 inside CTDI phantom.

Examining the TL samples with low energy X-rays and low-radiation dosimetry is crucial; hence, the use of a mammography application in our study. The TLD samples were irradiated by a GE-Senographe Pristina mammography machine. They were placed on an American College of Radiology (ACR) Accreditation phantom manufactured by CIRS Tissue Simulation & Phantom Technology, Model 15 [24] (see Figure 3). 

The samples were exposed to three sets of doses, selecting different mAs to measure the doses delivered to the TLD samples—to which end, a RaySafe X2 Mam detector calibrated at the KFSH&RC SSDL with an uncertainty of 4% [25] was placed beside the TLD samples (see Figure 3). The mammography machine was operated at a voltage of 28 kVp, and the selected mAs values were 45, 90, 135 and 180. A rhodium/silver (Rh/Ag) target/filter combination was selected for radiation exposure, and a peddle size of 24 mm × 29 mm was selected.

#### 2.3.3. Computed Tomography (CT)

The thermoluminescent dosimeters were exposed using a GE Revolution CT machine. The prepared samples were placed in a CTDI phantom manufactured by Sun Nuclear Tissue Simulation & Phantom Technology, Florida, USA [26]. The irradiation was performed using axial mode, a large focal spot and 120 kV with different tube currents of 185, 370 and 740 mA. The total radiation time was 0.5 s, and one image per rotation was selected with a detector coverage of 16 cm. The dose was measured via a RaySafe X2 CT Probe calibrated at SSDL of KFSH&RC with an overall uncertainty of 3.8% [25]. The pen dosimeter was placed in the center of the CTDI phantom for each selected current of 185, 270 and 740 mA to read the dose, and then TLD samples were placed (see Figure 3).

### 2.4. TL Reading and Annealing

A Harshaw 5500 reader was used to read the TL glow-curve-prepared dosimeters after irradiation from the different applications considered. The selected setting of the Time Temperature Profile (TTP) in the winrems operating software was 10 °C/s as the heating rate, the preheat time was 50 °C, and the reading time to reach maximum temperature of 400 °C was 37.66 s [27].

Annealing is an additional procedure that differs from reading. It is used to remove any residual signal or doses from the samples. This is performed using two ovens, set to 400 and 100 °C, where the second oven is used for constant cooling down. The samples were annealed in the first oven for 4 min, then moved to the second oven to cool down at a constant rate. Furthermore, the samples were placed outside for 20 min to cool down at room temperature. Finally, the samples were placed on the Harshaw 5500 rotator plate and annealed/read to record the residual signal.

## 3. Results and Discussion

### 3.1. TL Scanning Electronic Microscopy (SEM), Energy Dispersive X-ray Analysis (EDS)

**Figure 4:**
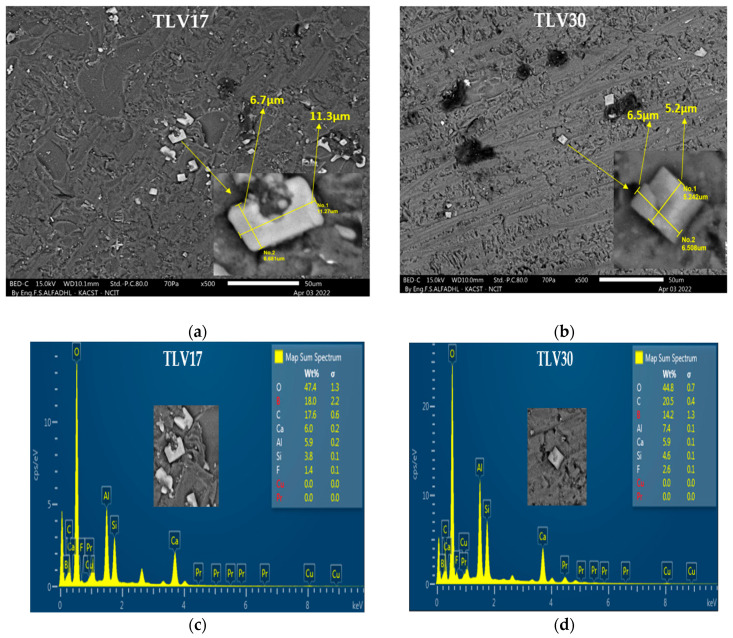
(**a**,**b**) SEM images and (**c**,**d**) elemental analysis EDS composition spectra for TLV17 and TLV30 samples.

The SEM analysis for the TLV17 dosimeter, as shown in Figure 4a,b, illustrates the microstructure and surface crystallization, from which it can be clearly seen that there is a higher density of crystals, the largest size of which is 6.7 × 11.3 µm^2^, whereas TLV30 showed fewer crystals that were smaller in size, at 6.5 × 5.2 µm^2^. In addition, the EDS analysis, as shown in Figure 4c,d for both TLV samples, confirms the existence of the initial elements (B, O, Al, Si and Ca) that originally used in the preparation of the samples TL17 and TLV30.

### 3.2. TL Glow Curves of Samples

**Figure 5:**
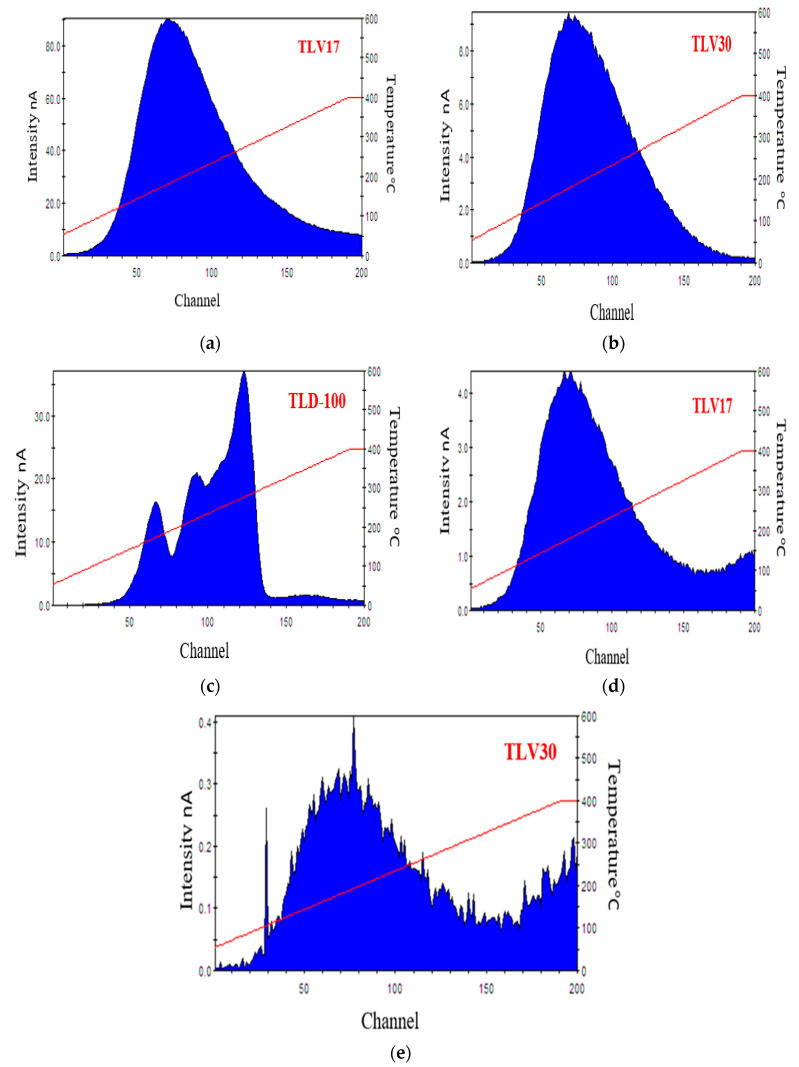
(**a**–**c**) The glow curves for the TLD samples at a dose of 50 mGy at 120 kVp. (**d**,**e**) The glow curves from a 2 mGy dose from a mammography machine at 28 kVp.

**Figure 6:**
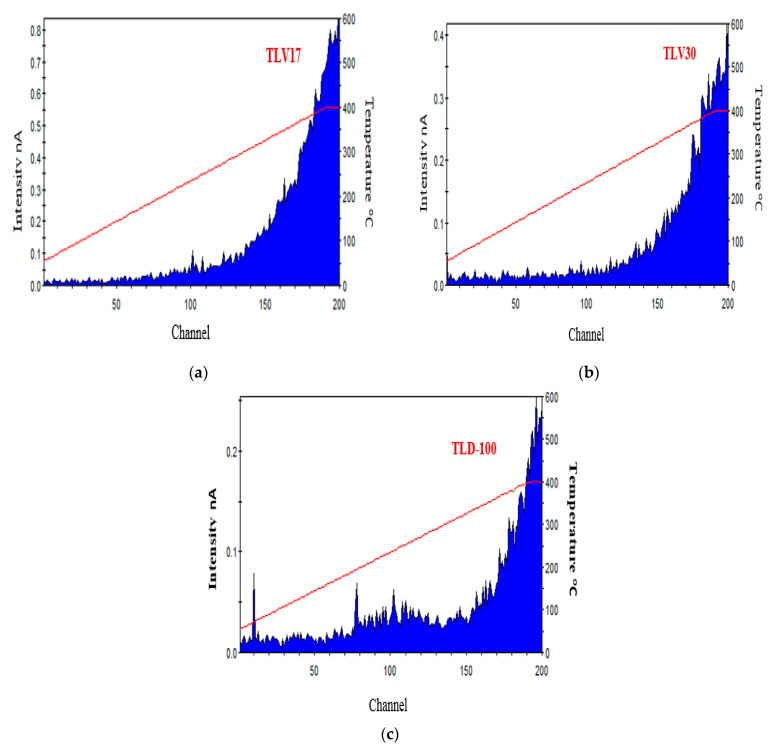
(**a**–**c**) The glow curves for the TLD samples without a radition dose.

The glow curve for the TL sample is an important dosimetric property as it indicates whether the sample can be used for radiation dosimetry purposes or otherwise [28]. Figure 5 shows one example of TL glow curves data of TLV17, TLV30 and TLD-100 that were irradiated at high and low doses. Higher TL sensitivity of TLV17 compared with both TLV30 and TLD-100 was observed. The observed formed dense crystals with large dimension in the TLV17 sample may be incorporated to the enhanced TL property, which is consistent with our previous work [18]. The glow curves for the TL samples that express the zero radiation dose/residual dose are illustrated in Figure 6. TLD-100 recorded the lowest residual dose, and TLV17 had the highest one.

### 3.3. Dosimetric TL Properties

#### 3.3.1. Dose Response and Dose–Response Linearity from SSDL Irradiation

The results for the TL samples that were irradiated by the SSDL ^137^Cs γ-ray and diagnostic X-ray with tube voltages of 40, 80, 100, 120 and 150 kVp, at doses ranging from 5 to 70 mGy are reported in detail in the Table 2 and Table 3.

**Figure 7:**
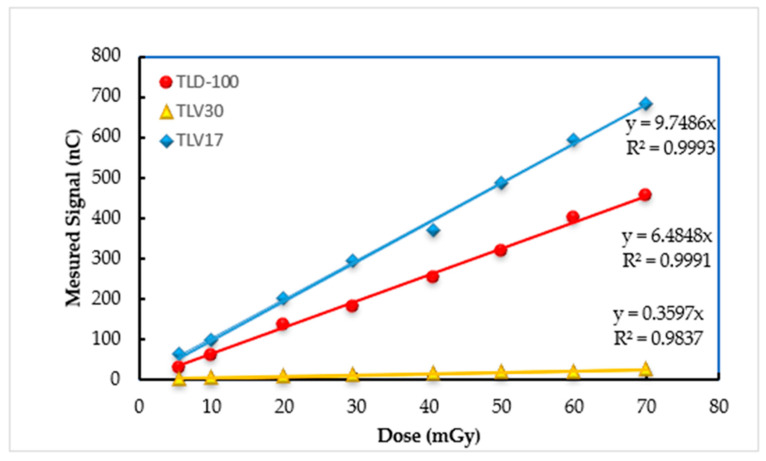
The dose–response linearity for TLV17, TLV30 and TLD-100 from SSDL 450 Ci of ^137^Cs.

**Figure 8:**
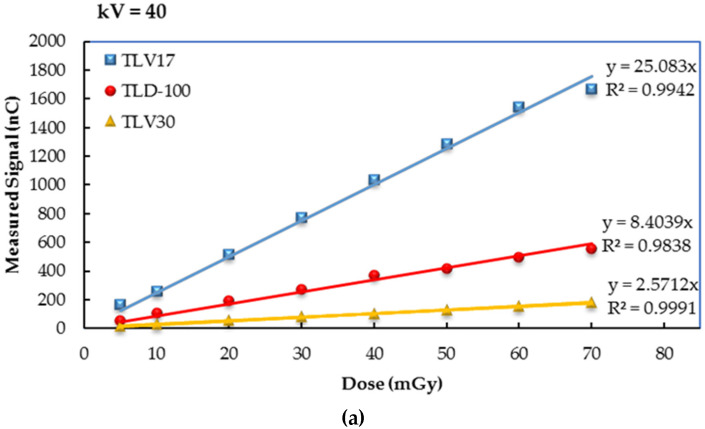

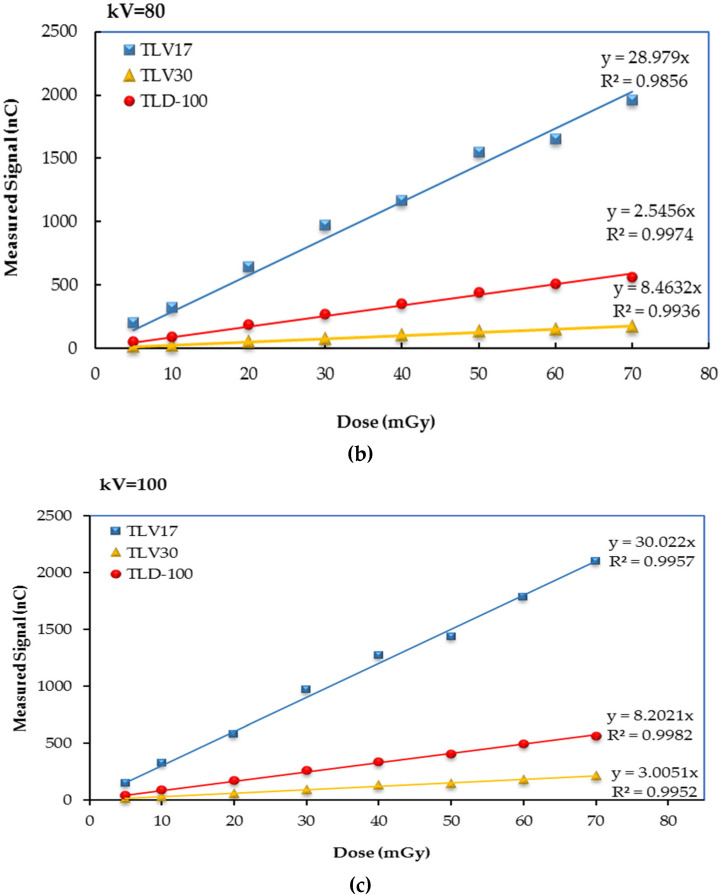

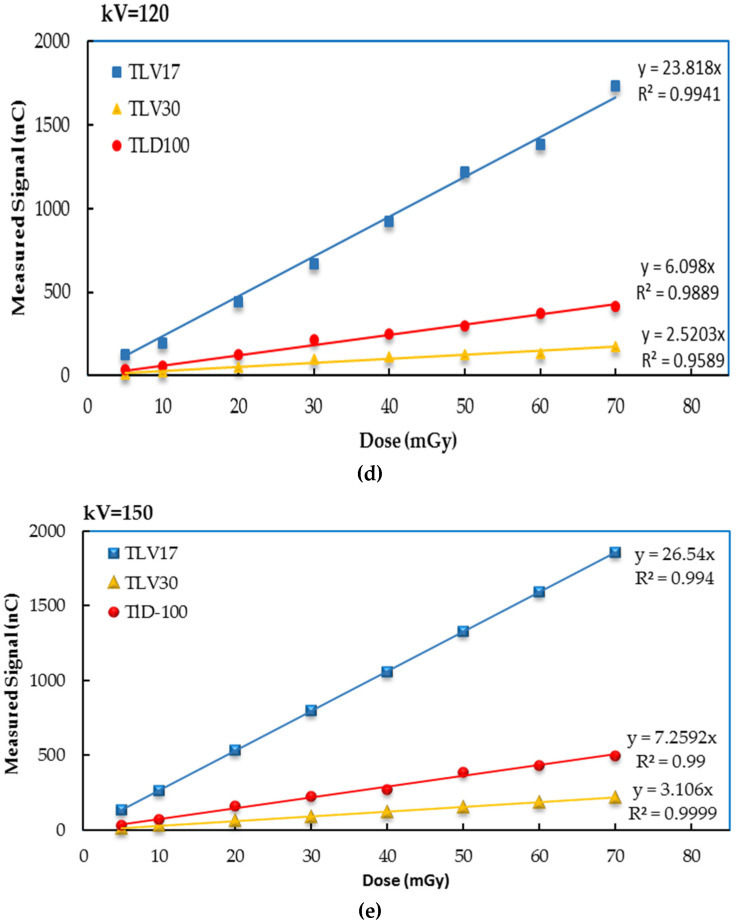
The dose–response linearity for TLV17, TLV30 and TLD-100 from SSDL daignostic X-ray beams. (**a**) KV = 40, (**b**) KV = 80, (**c**) KV = 100, (**d**) KV = 120, (**e**) KV = 150.

The results show clearly that TLV17 and TLV30 dosimeters have, respectively, the highest and lowest sensitivity compared with TLD-100. This is because the TL response was influenced by the crystal sizes and its crystallinity percentage in the amorphous state. All the dosimeters showed good dose–response linearity with a regression coefficient R^2^ ≥ 0.99; (see Figure 7 and Figure 8).

#### 3.3.2. Dose Linearity from Mammography

**Figure 9:**
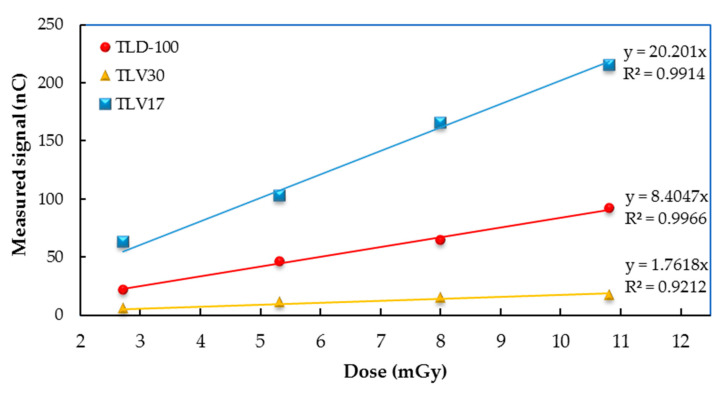
TLV17, TLV30 and TLD-100 radition dose–response linearity results from mammography.

The results of reading the TL dosimeters after irradiation via the GE Pristinia mammography machine are reported in Table 4. The setting of the mammography machine were as follows: kVp = 28; mAs (45, 90, 135 and 180). The dose for each selected mAs was measured via a RaySafe detector with values of 2.72, 5.32, 8 and 10.8 mGy. The TLV17, TLV30 and TLD-100 radiation dosimeters showed good linearity in terms of the dose response. Furthermore, as can be seen in Figure 9, the sensitivity of the TL samples in mammography is consistent with the results obtained with the SSDL with TLV17 and TLV30 samples, exhibiting, respectively, the highest and lowest sensitivities.

#### 3.3.3. Dose Linearity from CT Irradiation

**Figure 10:**
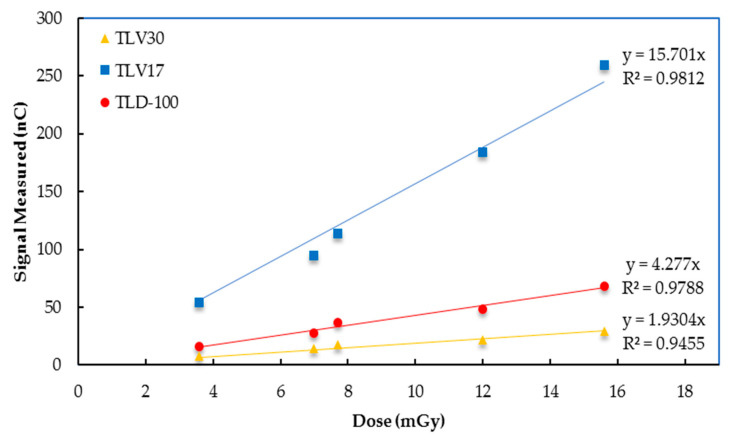
TLV17, TLV30 and TLD-100 CT radition dose–response linearity results.

The results of exposing the TL samples to three different doses by increasing the mA at each scan with a constant kVp of 120, a scanning time of 0.5 s, and by placing the TL samples in the center of CTDIvol phantom showed good dose–response linearity with coefficients of determination (R^2^) for TLV17, TLV30 and TLD-100 of 0.997, 0.98 and 0.998, respectively. In addition, the TLV17 dosimeter showed the highest dose response, greater than TLD-100 and TLV30, with the latter having the lowest dose response; see Figure 10.

CTDIcentre body phantom (32 cm diameter) was measured using a RaySafe pencil detector, TLV17, TLV30 and TLD-100 (see Table 5). The percentage errors for TLV17, TLV30 and TLD-100, mA = 185 compared with the RaySafe pencil detector were 13%, 19% and 2.8%, respectively. For mA = 370, the percentage errors were 17, 9.4 and 6%, respectively. Finally, for mA = 740, the percentage errors were 15%, 12.8% and 11%, respectively. It can be noted that TLV17 and TLV30 can be used in CT for dosimetry; nevertheless, further calibration procedures are required to obtain more accurate dosimetry.

#### 3.3.4. Reproducibility

The reproducibility of the TL signal was evaluated by irradiating each sample four times at the same dose and reading it. The standard deviation (STDEV), the relative STDEV, and the standard error of the results were calculated, which should be less than 5% [29]. In this study, the samples were exposed to a dose of 40 mGy from the 450 Ci ^137^Cs source and read after 5 min.

The calculated reproducibilities of the TL dosimeters were determined as shown in Table 6 after exposing the sample to a 40 mGy dose from a 450 Ci ^137^Cs source four times. The results of which showed that TLV17, TLV30 and TLD-100 had standard errors of 1.28, 1.95 and 0.75%, respectively, which are less than 5%, meaning that they have an acceptable reproducibility [29].

#### 3.3.5. Minimum Detectable Dose (MDD)

The minimum detectable dose is considered to be one of the most important dosimetric properties of a TL dosimeter [30,31]. It can be defined as the lowest radiation detection level of a prepared dosimeter or the threshold value of the sensed dose by the TL materials prepared. In terms of value, *MDD* is very similar to background signals [32].

(1) $$MDD=\left(B^{*}+2\;\sigma_{B}\right)\;\mathrm{CF}$$

where B* is the mion of thermoluminescence glow curves in quartz extracteal dose annealed four times, which were TLV17 = 3.6 nc, TLV30 = 1.95 nc and TLD-100 = 1.45 nc. *σ_B_* is the standard deviation of the four residual doses for TLV17, TLV30 and TLD-100, which were 0.36, 0.39 and 0.16, respectively. The average calibration factor (CF) for the TLD samples irradiated with ^137^Cs from Table 2 were TLV17 = 0.1 mGy/nc, TLV30 = 2.5 mGy/nc and TLD-100 = 0.16 mGy/nc. Using Equation (1), the MDD for TLV17 = 0.4 mGy, TLV30 = 6.8 mGy and TLD-100 = 0.3 mGy.

#### 3.3.6. Energy Dependence

The relative dose response *S’(E)* is defined as the ratio of detector response (*DR*) divided by the delivered dose from ^137^Cs [8,33]. The energy dependence of the TLD samples was investigated by irradiating them at 20 mGy using a SSDL 450 Ci ^137^Cs source and diagnostic radiology X-ray beams at different tube voltages of 40, 50, 60, 80, 100, 120, 150 and 250 kVp with effective energies of 26.1, 36.3, 41.8, 47.3, 61, 119 and 210 keV, respectively [23].

(2) $$S\prime (E)=\frac{\;\;{\left(\frac{DR}{k_{air}}\right)}_{E}}{\;\;\;\;\;\;\;\;\;{\left(\frac{DR}{K_{air}}\right)}_{137_{\mathrm{Cs}}}}$$

**Figure 11:**
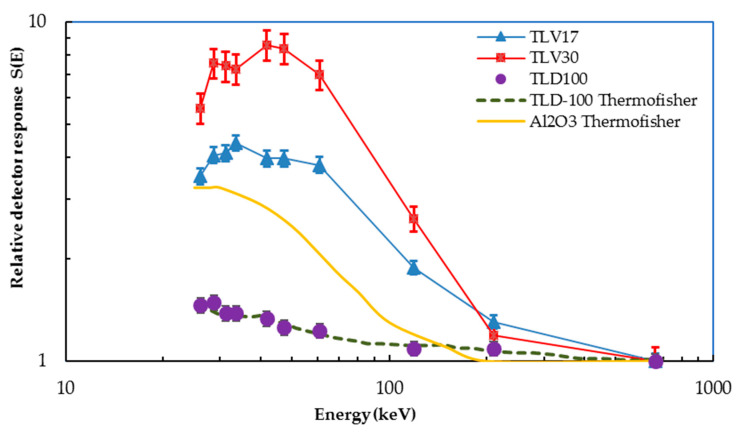
Energy dependence of TLV17, TLV30 and TLD-100.

By using Equation (2), the relation between the photon energies (keV) and relative detector response is plotted in Figure 10. It can be noticed that TLV30 has the highest energy dependence, whilst TLD-100 has the lowest. The plotted curve of TLD-100 (Harshaw, Waltham, MA, USA) irradiated in KFSH&RC-SSDL is consistent with the Harshaw TLD-100 curve published by Thermofisher [34]. Comparing TLV17 and TLV-30 curves with the AL_2_O_3_:C curve published by Thermofisher, it may be noted that they show similar behaviors, which could be a result of the effect of Al_2_O_3_ used in a mixture of the TLV17 and TLV30 samples as shown in Figure 11.

All dosimeters are calibrated using a ^137^Cs γ-source, and the energy dependence correction factor is implemented in the dose calculation algorithm. This is particularly important at lower energies, such as in mammography, where the energy response is higher. The overresponse of sample TLV17 is often associated with the increased photoelectric effect, which has a fourth to fifth power cross-section dependence on atomic number and Z as well as an approximately cubic inverse dependence on energy [35].

#### 3.3.7. Fading

To estimate the fading of the TL signal, the samples were irradiated at 10 mGy using the ^137^Cs gamma irradiator and evaluated after 5 min with the heating rate = 10 °C/s. This is the average elapsed time between the irradiation of the dosimeters and their subsequent evaluation in our study. This procedure was repeated, and the TL dosimeters were read with a heating rate of 10 °C/s after 1, 3, 8 and 28 days. The samples were stored in a dark drawer with a constant room temperature for each procedure. 

We observed that TLV17 and TLV30 each showed a steep decrease in dose after the first day with losses of 40% and 70%, respectively, whereas TLD-100 showed only a 9% loss. For TLV17 and TLV30, the steep signal decrease in the first day is due to shallow traps, which can be released more easily through thermal stimulation compared with traps with higher activation energies. High-temperature peaks are more stable than low-temperature peaks [33]. For TLD-100, the results are due to the fading of 50% in the first peak [36]. 

**Figure 12:**
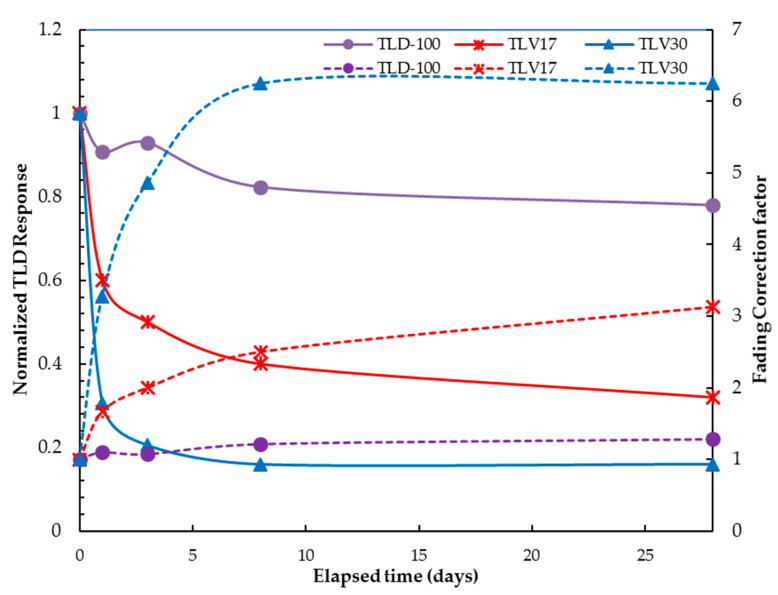
Fading (continues curves) and fading correction factor (dashed curves) of TLV17, TLV30 and TLD-100.

From day 1 to day 28, the fading in TLV17 and TLV30 were more stable, showing only slight decreases of 28% and 14.56%, respectively, compared to 13% for TLD-100, which is almost equal to the fading curve established by Thermofisher [34]; see Figure 12. An overall fading correction factor, calculated as the inverse of the fading (normalized TLD response) as shown in Figure 12, must be implemented in the dose calculation for greater accuracy [37]. In the future, further improvements and investigations of TLV17 and TLV30 should be pursued to reduce the fading factor as much as possible.

## 4. Conclusions

The thermoluminescence TLV17 and TLV30 dosimeters prepared from B_2_O_3_-CaF_2_-SiO_2_-Al_2_O_3_ doped with Cu and Pr atoms and the Thermofisher Harshaw TLD-100 used for comparison were examined in KFSH&RC-SSDL using 450 Ci ^137^Cs γ-rays, X-rays (tube voltage 40–150 kV), a dose range from 5–70 mGy and with two important radiology applications—namely, a CT dose range of 6–24 mGy and mammography with a dose range of 2.72–10.8 mGy. SEM and EDS analyses were performed on the synthesized TLV17 and TLV30 dosimeters, where the SEM images confirmed the existence of crystals, and the EDS spectra confirmed the presence of the elements originally used for preparation. 

The exposure of samples in different radiation applications showed observable glow peaks at 400 with a heating rate of 10 °C/s for all mentioned dose ranges. However, TLV17 had the highest sensitivity, and TLV30 had the lowest, whereas TLD-100 was in-between. This is because TLV17 contained a higher quantity of crystals that were larger in size than those in TLV30 as confirmed from the SEM images. In addition, the TLV17 and TLV30 dosimeters showed good dose responses and linearities similar to TLD-100, with coefficients of determination (R^2^) ≥ 0.99. 

The reproducibility for TLV17 and TLV30 had percentage errors of 1.28% and 1.95%, respectively, which are less than the recommended 5% and, therefore, acceptable as with TLD-100. The results of the fading investigations showed that, in general, TLV17 had less fading than TLV30. Both samples showed a steep decrease of TL signals after the 1st day, which then became stable with a slight decrease until the 28th day. Therefore, it is recommended that the TL dosimeters be read after 24 h, in the same way as TLD-100. The energy dependence of TLV30 was the highest, followed by TLV17, whereas TLD-100 had the lowest energy dependence. The energy dependence curves for TLV17 and TLV30 showed similar behaviors to the Al_2_O_3_:C curve published by Thermofisher, which could be due to the presence of the Al_2_O_3_ used in preparing the TLV17 and TLV30. 

The minimum dose detectability (MDD) for TLV17 = 0.58 mGy, TLV30 = 6.8 mGy and TLD-100 = 0.3 mGy. Overall, TLV17 showed better results than TLV30, and, as a result, we suggest its use in diagnostic radiology dosimetry with implementing the required energy and fading factors. Future improvement with regard to reducing the fading is recommended, however.

## Figures and Tables

**Table 1:** Samples, compositions and preparation methods of the TL dosimeters used in the present study.

TLD Samples	Composition	Preparation
TLV17	10 CaF_2_: 10 SiO_2_: 10 Al_2_O_3_: 70 B_2_O_3_: (Cu and Pr: 1500 ppm)	15 h heat-treated after melt-quenching
TLV30	10 CaF_2_: 10 SiO_2_: 10 Al_2_O_3_: 70 B_2_O_3_: (Cu and Pr: 1500 ppm)	2 h heat-treated after melt-quenching
TLD-100	LiF: Ti, Mg	Crystals from Thermofisher Inc. (Waltham, MA, USA)

**Table 2:** TL dose results from ^137^Cs irradiation from SSDL.

TLD Samples	TLV17		TLV30		TLD-100	
Air Kerma (mGy)	Reading (nC)	Calibration Coefficient (mGy/nC)	Reading (nC)	Calibration Coefficient (mGy/nC)	Reading (nC)	Calibration Coefficient (mGy/nC)
5	65	0.08	3	1.83	29	0.19
10	99.44	0.1	4.7	2.13	61	0.16
20	201	0.1	8.5	2.3	135	0.15
30	293	0.11	11	2.7	180	0.16
40	371	0.1	16	2.6	253	0.16
50	488	0.1	18	2.61	320	0.16
60	594	0.1	20	2.78	403	0.15
70	683	0.1	25	2.96	455	0.15
Average		0.1		2.52		0.16
STDEV		0.0074		0.4		0.0014
Relative STDEV		7.4%		15.7%		8.6%

**Table 3:** TL dose results from X-ray irradiation from SSDL.

		kV = 40 keV = 26.1		kV = 80 keV = 36.3		kV = 100 keV = 41.8		kV = 120 keV = 47.3		kV = 150 keV = 61	
Sample	Air Kerma (mGy)	Reading (nC)	Cal. Coefficient (mGy/nC)	Reading (nC)	Cal. Coefficient (mGy/nC)	Reading (nC)	Cal. Coefficient (mGy/nC)	Reading (nC)	Cal. Coefficient (mGy/nC)	Reading (nC)	Cal. Coefficient (mGy/nC)
TLV17	5	169	0.03	205	0.02	149	0.03	127	0.04	132.5	0.04
	10	257	0.04	323	0.03	326	0.03	191.6	0.05	265	0.04
	20	514	0.04	640	0.03	578	0.03	441	0.05	532	0.04
	30	771	0.04	969	0.03	967	0.03	668	0.04	797	0.04
	40	1245	0.03	1167	0.03	1268	0.03	924	0.04	1060	0.04
	50	1285	0.04	1550	0.03	1435	0.03	1220	0.04	1329	0.04
	60	1542	0.04	1650	0.04	1786	0.03	1386	0.04	1592	0.04
	70	1669	0.04	1959	0.04	2098	0.03	1736	0.04	1857	0.04
Average		0.04			0.03		0.03		0.04		0.04
STDEV			0.0040		0.004		0.002		0.0041		0
Relative STDEV			10.7%		11.8%		5.3%		9.3%		0.2%
TLV30	5	16.3	0.307	16	0.31	15	0.33	11	0.45	15.625	0.32
	10	28	0.357	26	0.38	29	0.34	24	0.42	31.25	0.32
	20	53	0.377	50	0.40	52	0.38	46.6	0.43	61.55	0.32
	30	78	0.385	72.4	0.41	90.4	0.33	97	0.31	92.8	0.32
	40	103	0.388	104	0.38	128	0.31	115	0.35	125	0.32
	50	128	0.391	132	0.38	144	0.35	125	0.40	154.35	0.32
	60	153	0.392	153	0.39	180	0.33	134	0.45	186.55	0.32
	70	180	0.389	175	0.40	213	0.33	176	0.40	218	0.32
Average		0.37			0.38		0.34		0.40		0.32
STDEV			0.03		0.032		0.022		0.05		0.002
Relative STDEV			8.2%		8.5%		6.5%		13.4%		0.6%
TLD-100	5	53	0.09	50.2	0.10	41	0.12	35	0.14	34	0.15
	10	106	0.09	89.6	0.11	86	0.12	56	0.18	68	0.15
	20	191	0.10	185	0.11	175	0.11	124	0.16	159	0.13
	30	271	0.11	266	0.11	259	0.12	216	0.14	227	0.13
	40	372	0.11	349	0.11	333	0.12	248	0.16	272	0.15
	50	418	0.12	440	0.11	405	0.12	295	0.17	386	0.13
	60	497	0.12	506	0.12	493	0.12	372	0.16	431	0.14
	70	557	0.13	565	0.12	565	0.12	412	0.17	499	0.14
Average			0.11		0.11		0.12		0.16		0.14
STDEV			0.011		0.006		0.004		0.014		0.01
Relative STDEV			9.7%		5.4%		3%		8.8%		6.6%

**Table 4:** TLV17, TLV30 and TLD-100 mammography radiation dose results.

TL Samples		TLV17	TLV30	TLD-100
mAs	Dose (mGy)	Reading (nC)	Reading (nC)	Reading (nC)
45	2.72	63.2	5.4	22
90	5.32	103	11	46
135	8	166	15	65
180	10.8	215	17.4	92
kVp = 28 Average Cal. Cof.		0.048 mGy/nC	0.53 mGy/nC	0.12 mGy/nC

**Table 5:** TLV17, TLV30 and TLD-100 CT radiation dose results.

Samples	RaySafe Detector	TLV17		TLV30		TLD-100	
mA	Dose CTDIcent. (mGy)	Reading (nC)	Dose CTDIcent. (mGy)	Reading (nC)	Dose CTDIcent. (mGy)	Reading (nC)	Dose CTDIcent. (mGy)
185	3.6	54	3.2	8	4.4	16	3.36
250	5.2	95	5.7	14	7.56	28	5.9
370	7.7	114	6.84	18	9.7	37	7.8
550	11.4	184	11.04	22	11.88	48	10.1
740	15.6	259	15.54	29	13.34	68	14.3
kV = 120 Average Cal. Cof.		0.06 mGy/nC		0.46 mGy/nC		0.21 mGy/nC	

**Table 6:** The reproducibility of the TL samples.

TL Samples	Reading 1 (nC)	Reading 2 (nC)	Reading 3 (nC)	Reading 4 (nC)	Average Reading (nC)	STDEV	Relative STDEV (%)	Standard Error (%)
TLV17	413	426	405	436	414.7	10.59	2.56	1.28
TLV30	18.5	19.8	18.17	18.4	18.8	0.73	3.9	1.95
TLD-100	255	255	250	253	253	2.88	1.14	0.57

## Data Availability

The dataset generated during the current study is available from th corresponding author on reasonable request.
